# Progression-Aware and Explainable CNN–Transformer Framework for Multiclass Alzheimer’s Disease Staging Using MRI

**DOI:** 10.3390/diagnostics16040593

**Published:** 2026-02-16

**Authors:** Khalaf Alsalem, Murtada K. Elbashir, Ahmed Omar Alzahrani, Mohanad Mohammed, Mahmood A. Mahmood, Tarek Abd El Fattah

**Affiliations:** 1 Department of Information Systems, College of Computer and Information Sciences, Jouf University, Sakaka 72388, Saudi Arabia; mkelfaki@ju.edu.sa (M.K.E.); mamahmood@ju.edu.sa (M.A.M.); 2 Department of Information Systems and Technology, College of Computer Science and Engineering, University of Jeddah, Jeddah 21959, Saudi Arabia; aoalzahrani@uj.edu.sa; 3 Discipline of Statistics, School of Agriculture and Science, University of KwaZulu-Natal, Durban 4041, South Africa; mohammedm1@ukzn.ac.za; 4 Department of Information Systems, Faculty of Information Technology, Misr University for Science and Technology, Cairo 3237101, Egypt; tarek.mohamed@must.edu.eg

**Keywords:** Alzheimer’s disease, MRI, multiclass staging, CNN–Transformer, ordinal learning, interpretability

## Abstract

**Background:** Alzheimer disease (AD) is a neurodegenerative condition that progressively develops structural changes in the brain, resulting in different stages of severity, which makes accurate multiclass classification from magnetic resonance imaging (MRI) challenging. Despite promising outcomes of deep learning models, a great number of current methods disregard disease progression, suffer from evaluation leakage, or lack interpretability. **Objectives:** This paper introduces DeepAttentionADNet, a lightweight hybrid CNN–Transformer framework designed for multiclass staging of Alzheimer’s disease using MRI images. **Methods:** The proposed model integrates convolutional feature extraction with transformer-based global context modeling. To capture the ordered nature of disease severity, a progression-aware ordinal learning objective is proposed. Moreover, consistency regularization is utilized to enhance robustness by imposing consistent prediction with spatial perturbation. A leakage-free k-fold cross-validation protocol is adopted, in which data splitting is performed prior to augmentation. Also, to promote interpretability, token-level importance maps based on transformer embeddings are utilized to visualize spatial regions that were used to make classification decisions. **Results:** The experimental findings on a multiclass MRI dataset of Alzheimer disease demonstrate consistent and high performance across cross-validation folds (mean F1-score (0.991 ± 0.003) and AUROC (0.9998 ± 0.0002)), without losing transparency and progress awareness. **Conclusions:** The proposed framework provided a robust and interpretable method of Alzheimer disease severity classification using MRI.

## 1. Introduction

Alzheimer disease (AD) is a progressive and irreversible neurodegenerative condition, which is marked by constant deterioration of cognition, behavioral abnormality and progressive functional independence [1]. Being the most prevalent type of dementia, AD now represents over 55 million cases in the world, and it is expected to almost triple the number by 2050 because of population aging [2]. The early clinical manifestations of AD are difficult to identify because initial symptoms are similar to those of the normal aging process and other neurological disorders, leading to delayed diagnosis and missed opportunities for early intervention [3]. Magnetic resonance imaging (MRI) plays a crucial role in the diagnosis and follow-up of AD because it is non-invasive and has a high spatial resolution [4]. Structural MRI makes it possible to examine brain morphology in detail so that the clinician can identify cortical thinning, ventricular enlargement, and hippocampal atrophy, which are characteristic biomarkers of AD progression [5]. The increasing availability of large-scale datasets has facilitated the integration of machine learning and deep learning methods for the automated analysis of MRI scans to detect and classify diseases [6]. Recent developments in deep learning have made convolutional neural networks (CNNs) a paradigm in medical image analysis and continue to surpass conventional machine learning approaches when it comes to visual recognition tasks [7]. In the context of Alzheimer’s disease, CNN-based models have been widely applied to MRI data to identify pathological patterns and differentiate between disease stages. Nonetheless, a large number of current CNN-based methods face persistent challenges, including limited generalization due to small or imbalanced datasets, susceptibility to overfitting, and difficulty in capturing subtle inter-stage anatomical variations, particularly in early stages of the disease [6,8]. Nevertheless, the MRI-based classification of the AD still has several unaddressed issues. Publicly available datasets, including the Alzheimer’s Disease Neuroimaging Initiative (ADNI) tend to have a significant imbalance in their classes across disease stages, potentially leading to biases in the training and evaluation of the model [9]. Moreover, the lack of data diversity and absence of consistent preprocessing limits models’ ability to learn robust and generalizable representations [10]. In addition, the majority of the current literature focuses on binary classification (AD vs. cognitively normal) and neglects clinically significant intermediate conditions such as mild cognitive impairment (MCI) and very mild dementia, which are important in terms of diagnosis and disease monitoring in the early stages [11].

In addition to the challenges associated with data, most deep learning methods consider the stages of Alzheimer disease as discrete categories without regard to the fact that the progression of disease is an ordinal process. This may result in clinically plausible predictions including the wrongful classification of patients with advanced stages as cognitively normal. Recent papers have highlighted the necessity of considering the disease progression constraints and clinically guided learning goals into the deep learning models of neurodegenerative disorders [9]. Simultaneously, attention-based networks and transformer networks have become very powerful to capture long-range spatial interactions in medical images to provide better contextual reasoning, than local convolutional networks [12,13]. Nevertheless, they are not yet extensively applied in staging multiclass Alzheimer’s disease and issues of robustness and interpretability continue to be of concern. To address the imbalance of classes and make multiclass learning reliable, the Alzheimer Multiclass MRI Dataset proposed by Singhal (2022) [14] provides a balanced and expanded set of MRI images across four disease categories: Non-Demented, Very Mild Demented, Mild Demented, and Moderate Demented. This data is well-balanced in terms of disease stage, facilitating effective training and evaluation using deep learning models, and multiclass classification frameworks can be developed. Even though CNN–Transformer hybrid architectures, attention mechanisms, and interpretability techniques have all been investigated in the past for the analysis of Alzheimer’s disease, most studies either concentrate on classification accuracy or address these elements separately. Specifically, earlier multiclass approaches do not explicitly model the ordinal and progressive nature of Alzheimer’s disease severity; instead, they treat disease stages as separate categories. Furthermore, interpretability is frequently restricted to static attention visualizations without progression-aware analysis. Also, robustness to slice-level perturbations and the clinical plausibility of misclassifications are frequently overlooked. This paper presents DeepAttentionADNet, a CNN–Transformer model designed for multiclass Alzheimer’s disease classification using MRI images. The proposed solution extends traditional CNN models by incorporating convolutional feature extraction together with transformer-based global context modeling, enabling the capture of both local anatomical features and large-scale spatial relationships. A progression-aware ordinal learning objective is incorporated to better represent the ordered nature of Alzheimer’s disease severity. In addition, a consistency regularization strategy is used to enhance robustness through imposing consistent predictions in the presence of spatial perturbations that are typical of MRI data. A leakage-free k -fold cross-validation protocol is used to guarantee consistent and objective performance evaluation, with data partitioning performed before augmentation. Additionally, token representations based on the transformer module are also used to increase model interpretability, which gives an idea about which regions make the most significant contribution to the distinction of disease stages. The main contributions of this study are summarized as follows:

- We use progression-aware ordinal learning to directly model the ordering of severity of Alzheimer disease.
- We apply consistency regularization to enhance the robustness of the slice-level predictions of MRI images.
- We provide token-level interpretability analysis to improve transparency and trust in model decisions.
- We design an efficient CNN–Transformer architecture suitable for real-world MRI imaging applications.

## 2. Related Work

Deep learning (DL) and machine learning (ML) methodologies have been widely investigated over the past decade for the detection and classification of Alzheimer’s disease (AD). In this literature review section, we primarily focus on the works that develop automated neuroimaging data analysis frameworks, with common challenges including class imbalance, limited sample size, and the complexity of multiclass disease staging. Convolutional neural networks (CNNs) have emerged as a leading approach for Alzheimer’s disease diagnosis using MRI because of their ability to automatically learn hierarchical spatial features. Early studies demonstrated that stacked autoencoders and CNN-based models were effective in differentiating Alzheimer’s disease and mild cognitive impairment (MCI) from normal controls [15]. Likewise, 3D-CNN networks are suggested to explicitly process volumetric MRI data architectures that do not require manual feature extraction and perform well in classifying the stage of Alzheimer disease [16]. Despite these achievements, CNN-based models often struggle to detect subtle anatomical variations between adjacent disease stages, particularly in early-stage AD and MCI. Transfer learning has been broadly implemented to mitigate the problem of limited labeled neuroimaging data. Residual and plain convolutional neural network architecture were fine-tuned on MRI images [17], thereby leading to an increase in the diagnostic accuracy. Similarly, modified ResNet models were also used on 3D brain images to detect early-stages of Alzheimer’s disease, and they demonstrate a better feature representation capacity compared to training from scratch [18]. Although transfer learning enhances convergence and performance, such models remain dependent on convolutional operations mainly and do not have the capability to represent long-range spatial dependence. Other researchers have investigated the concept of multimodal learning to enhance diagnostic performance through combining complementary sources of information. Jin et al. [19] proposed multimodal cross-attention model that is used to jointly analyze the structural MRI (sMRI), fluorodeoxyglucose positron emission tomography (FDG-PET), and cerebrospinal fluid (CSF) biomarkers. Their method uses cascaded enhanced convolution and cross-modal attention to be aware of interactions between imaging and non-imaging data. Although multimodal approaches can improve diagnostic accuracy, they require multiple data modalities that are not always available in routine clinical practice. The imbalance of the classes is one of the significant obstacles to the classification of the Alzheimer disease, especially in the public datasets, e.g., in the ADNI, where intermediate stages of the diseases are not fairly represented. Falahati et al. [20] pointed out the imbalanced representation of the subjects in different categories of diseases and their effect on the training of the model. To solve this problem, several resampling methods have been proposed, including class reweighting, and oversampling. As Wen et al. [9] have shown that balancing training batches is more effective in enhancing recall of minority-class and the overall robustness. Parallel Data augmentation has been extensively applied to augment effective sample size. Augmentation methods such as rotation, flipping and scaling have been proven to enhance the CNN performance in imaging tasks [21]. More advanced methods, like anatomically consistent augmentation [22] and intensity variation elastic deformation [23], are meant to simulate natural variability of the morphology of the brain without compromising the anatomical validity. Even though it has clinical importance, multiclass Alzheimer disease classification has received rather lower attention in comparison with binary diagnosis. It is also challenging to differentiate between non-demented, very mild, mild and moderate stages of dementia because anatomical differences between these early stages of the disease are minimal [24]. According to Basaia et al. [25], conventional CNNs tend to misclassify both the stages of MCI and early AD, which indicates the necessity of the more sophisticated operation of feature extractors. To overcome such shortcomings, attention-based and hybrid architectures have been developed. Li et al. [26] proposed Enhanced Residual Attention Network (ERAN) to classify medical images, demonstrating the effectiveness of attention-enhanced residual feature extraction. The potential of self-attention mechanisms is shown by Brar et al. [27], proposed an interpretable deep learning framework based on inverted self-attention and a vision transformer for Alzheimer’s disease prediction using MRI images. Other classifications have further used CNNs together with sequential or temporal models, including CNN-LSTM models, to enhance diagnostic performance [28]. It has also been suggested that the granular feature integration technique can be used to capture coarse and fine-grained information at various levels [29]. More recently, Alorf [30] proposed a CNN–Transformer hybrid model in multiclass Alzheimer disease classification, which demonstrates the benefits of local convolutional feature enhancement with attention-based reasoning on a global scale.

Due to their capacity to identify long-range spatial dependencies in neuroimaging data, transformer-based and attention-driven architectures have recently drawn more attention for the classification of Alzheimer’s disease [13,27]. By combining local feature extraction and global contextual reasoning, several studies have shown that CNN–Transformer hybrid models and ensemble learning can perform better than purely convolutional architectures [30,31]. To improve interpretability and robustness in Alzheimer’s disease diagnosis, recent research has also investigated self-attention mechanisms, which allow for spatial relevance analysis of discriminative brain regions [25]. Additionally, CTM-Net, a hybrid CNN–Transformer model, is used to diagnose Alzheimer’s disease using MRI. A transformer with multi-head attention captures long-range dependencies across slices, while a channel-attention CNN extracts fine-grained local features from MRI slices [32].

Even though the existing studies demonstrate the potential of deep learning for Alzheimer’s disease classification, several limitations remain. Many methods ignore the ordinal nature of Alzheimer’s disease progression and treat disease stages as independent classes. Robustness to slice-level perturbations and interpretability of model decisions are also often overlooked. Furthermore, evaluation protocols in prior work are sometimes affected by data leakage or inconsistent validation strategies. These limitations motivate the development of progression-aware, robust, and interpretable deep learning frameworks for multiclass Alzheimer’s disease staging, which is the focus of the present study.

## 3. Methodology

### 3.1. Dataset Description

In this paper we utilized Alzheimer Disease Multiclass MRI dataset that is presented by Singhal (2022) [14]. This data set is designed to perform multiclass classification of the severity of Alzheimer disease (AD). The dataset consists of skull-stripped brain MRI images, and all the scans are preprocessed in a way that they remove non-brain tissues so that they are consistent and can be analyzed using deep learning. The images are classified into four clinically significant groups of disease progression levels: Non-Demented (12,800 images), Very Mild Demented (11,200 images), Mild Demented (9856 images) and Moderate Demented (6528 images). Even though the sample size of the individual classes differs, the categories are large enough to allow the successful training and testing of multiclass classification models. The sequential arrangement of these groups indicates the natural course of Alzheimer disease regarding the transition between normal cognition to moderate dementia which makes the dataset especially suitable for progression-conscious and ordinal learning models.

### 3.2. The Proposed Framework

**Figure 1:**
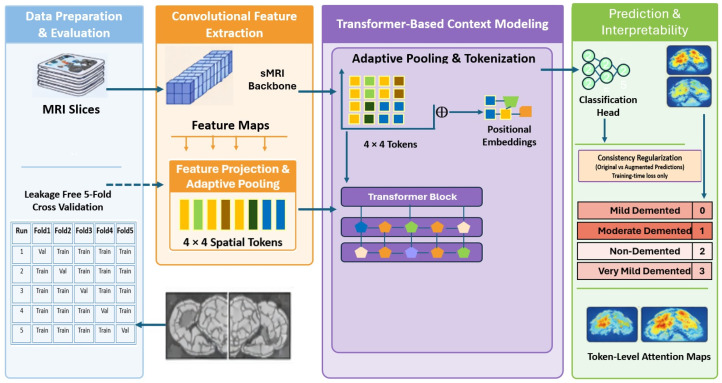
Overall architecture of the proposed DeepAttentionADNet framework.

This study proposes DeepAttentionADNet, a progression-aware and explainable hybrid CNN–Transformer model for classifying multiclass Alzheimer’s disease (AD) based on magnetic resonance imaging (MRI). The framework is designed to address the primary issues of AD staging, such as subtle inter-stage anatomical variation, the strength of slice-level predictions, and the need for interpretable model decisions. The proposed pipeline is composed of four key components, which are: (i) data preparation and leakage-free evaluation, (ii) convolutional feature extraction, (iii) transformer-based global context modeling, and (iv) progression-aware and consistency-regularized learning. A summary of the entire framework is depicted in Figure 1. A convolutional neural network (CNN) backbone is used to extract local feature representations out of MRI slices, which is then adaptively pooled and tokenized into fixed-length spatial tokens. This is then followed by using a transformer module to extract global contextual relationships on the tokens, with each transformer block operating multi-head self-attention to learn spatial dependencies, and a feed-forward network is used to refine features. The model is trained with a mix of cross-entropy loss, ordinal loss and prediction-level consistency regularization, on original and augmented inputs. Lastly, the framework produces progression-aware multiclass predictions along with interpretable token-level attention maps highlighting discriminative brain regions.

### 3.3. Data Preparation and Leakage-Free Evaluation Protocol

The preprocessed, skull-stripped 2D MRI slices are available from the Kaggle Alzheimer Multiclass MRI dataset. In this study, no extra skull-stripping or anatomical preprocessing was applied. The 2D MRI slices in the data set were pre-extracted; no additional slice selection or anatomical filtering was carried out for this study. All available slices were included in the dataset, which did not restrict the analysis to any specific anatomical level. Our evaluation protocol rigorously enforces dataset splitting before any augmentation, even though the dataset repository contains augmented images. During training, only online augmentations (rotation, horizontal flipping, and random resized cropping) are used; validation images only undergo resizing and normalization. By using this technique, it is guaranteed that no augmented version of the same image will appear in both the training and validation folds. The slice-Level MRI images are organized by subject and cross-validation splitting is performed before augmentation to prevent evaluation leakage. This way we can guarantee that no transformed version of the same slice is shared across folds. This slice-level independence assumption follows established practice in MRI-based Alzheimer classification studies using 2D image datasets.

The MRI images are classified into four diagnostic categories based on the stages of severity of Alzheimer disease Non-Demented, Very Mild Demented, Mild Demented, and Moderate Demented. A leakage-free k-fold cross-validation protocol is used to obtain unbiased performance evaluation. Dataset splitting is performed before data augmentation, so that no data augmentations of the same image can exist in training and validation folds. The methods of data augmentation (random cropping, rotation, and horizontal flipping) are only used in the training folds and validation images are only resized and normalized. This evaluation plan eliminates the probability of information leakage and gives a good model generalization in unseen data. The cross-validation and augmentation strategy can be formally expressed as follows:

Cross-validation:

$$D=\overset{K}{\underset{k=1}{⋃}}D_{k},\;D_{i}\cap D_{j}=⌀\;\mathrm{for}\;i\neq j,$$

$$D_{\mathrm{train}}^{\left(k\right)}=D\setminus D_{k},\;D_{\mathrm{val}}^{\left(k\right)}=D_{k}.$$

Augmentation:

$$\tilde{x}=T\left(x\right),\;T\in \left\{\mathrm{crop},\;\mathrm{rotate},\;\mathrm{flip}\right\}.$$

where D is the complete MRI dataset, $D_{k}$ is the subset corresponding to the *k*-th fold, and *K* is the total number of folds used in cross-validation. For each fold *k*, $D_{\mathrm{train}}^{\left(k\right)}$ and $D_{\mathrm{val}}^{\left(k\right)}$ are the training and validation sets, respectively. T(·) is the stochastic data augmentation function applied only to training samples, generating an augmented image $\tilde{x}$ from the original image *x*.

### 3.4. Convolutional Feature Extraction

The convolutional backbone used in feature extraction is based on ResNet-18 that has been trained on ImageNet. The last fully connected layer is removed to maintain spatial feature maps. CNN learns hierarchical representations, which describe local anatomical features used in the anatomical structures of Alzheimer disease. The extracted feature maps are adaptively pooled to a fixed spatial resolution of 4×4, enabling consistent tokenization regardless of input image size. A convolutional projection layer of size 1×1 is then used which will project the pooled features into a reduced dimensional embedding space that can be fed through a transformer. The feature extraction and tokenization process can be formally expressed as:

$$F=f_{\mathrm{CNN}}\left(x\right),\;F\in R^{C\times H\times W}$$

$$F^{\prime }=\mathrm{Pool}\left(F\right),\;F^{\prime }\in R^{C\times 4\times 4}$$

$$Z=W_{1\times 1}*F^{\prime },\;Z\in R^{D\times 4\times 4}$$

$$T=\mathrm{reshape}\left(Z\right)\in R^{16\times D}$$

where *x* is the input MRI slice, and $f_{\mathrm{CNN}}\left(\cdot \right)$ is the convolutional backbone (ResNet-18 without the final fully connected layer). $F\in R^{C\times H\times W}$ are the spatial feature maps extracted by the CNN, where *C* is the number of channels, and *H* and *W* are the spatial dimensions. Pool(·) is the adaptive average pooling operation that resizes feature maps to a fixed spatial resolution. $F^{\prime }\in R^{C\times 4\times 4}$ are the pooled feature maps with fixed spatial size. $W_{1\times 1}$ is the learnable 1×1 convolutional projection weights. $Z\in R^{D\times 4\times 4}$ are the projected feature maps, where *D* is the embedding dimension. $T\in R^{N\times D}$ are the set of spatial tokens obtained by reshaping Z, with N=16 tokens corresponding to the 4×4 spatial grid, each token $t_{i}\in R$ representing a local MRI region.

### 3.5. Transformer-Based Global Context Modeling

In adaptive pooling, after reshaping the projected feature maps, the resultant maps are converted into a series of spatial tokens, with each token representing a specific section of a slice of the MRI. Position embeddings are made learnable so that the spatial information can be retained. To model long-range dependencies between tokens, a lightweight transformer block based on multi-head self-attention and feed-forward layers is used. This allows the network to learn global contextual relations across spatial regions that might be challenging to learn with convolutional operations only. The outputs in the form of tokens are pooled together through mean pooling and sent to the classification head to predict the disease stage. The transformer-based global context modeling can be formally expressed as follows:

$$T_{0}=T+P$$

$$\mathrm{MSA}\left(T_{0}\right)=\mathrm{softmax}\;\left(\frac{QK^{⊤}}{\sqrt{D}}\right)V$$

$$T_{1}=T_{0}+\mathrm{MSA}\left(T_{0}\right)$$

$$T_{2}=T_{1}+\mathrm{FFN}\left(T_{1}\right)$$

$$z=\frac{1}{N}\sum_{i=1}^{N}t_{i},\;\hat{y}=f_{\mathrm{cls}}\left(z\right)$$

where $T_{0}=T+P$ is the position-aware token embeddings. $\mathrm{MSA}(T_{0})$ is the multi-head self-attention mechanism that models long-range dependencies between tokens. Q, K, V are the query, key, and value matrices computed from $T_{0}$ using learnable linear projections. FFN(·) is the feed-forward network applied within the transformer block for feature refinement. $T_{1}$ and $T_{2}$ are the intermediate and final token representations after attention and feed-forward refinement, $z\in R^{D}$ is the global image representation obtained via mean pooling over all tokens. $f_{\mathrm{cls}}\left(\cdot \right)$ is the classification head composed of normalization and fully connected layers. $\hat{y}$ is the predicted Alzheimer’s disease stage. All these elements allow the proposed framework to represent the local anatomical detail and the global contextual relationship and make the interpretation possible using the spatial token representations.

### 3.6. Progression-Aware Ordinal Learning

The stage of Alzheimer disease has natural ordinal nature, which progresses from non-demented to moderate stages of dementia. To fit this clinical prior, the framework proposes a progression-aware ordinal loss in addition to the existing cross-entropy loss. The ordinal loss penalizes the difference between the expected label of the disease that is calculated as the probability-weighted average of the indices of classes and the actual stage of the disease. This promotes predictions which observe disease severity ordering and minimizes clinically implausible prediction errors between distant stages. The progression-aware ordinal loss can be defined as follows:

p=softmax(z)

$$\hat{s}=\sum_{c=0}^{C-1}c\cdot p_{c}$$

$$L_{\mathrm{ord}}={\left(\hat{s}-y\right)}^{2}$$

The model logit is denoted by z and the predicted probability for class *c* is denoted by $p_{c}$. The ground truth disease stage label is given by y∈{0,…,C−1}, where *C* represents the total number of classes. The ordinal loss promotes the prediction that takes into account the natural sequence of stages of Alzheimer disease.

### 3.7. Consistency Regularization for Robust Prediction

MRI slice-level predictions are prone to small spatial perturbations. A consistency regularization strategy is implemented to enhance robustness during training. The network is trained to generate original image probability distributions that are similar to perturbed image probability distributions (e.g., horizontally flipped images). Consistency regularization is achieved by reducing the mean squared error of softmax outputs between image views where the pair view of the image is taken. It is this constraint that leads to stable representations and enhances the generalization in the event of common imaging variations. The consistency regularization constraint can be expressed as follows:

$$p=\mathrm{softmax}\;\left(f_{\theta }\left(x\right)\right),\;\tilde{p}=\mathrm{softmax}\;\left(f_{\theta }\left(\tilde{x}\right)\right)$$

where *x* is the input MRI slice and $\tilde{x}$ is the perturbed version of *x* (e.g., horizontal flip). $f_{\theta }\left(x\right)$ is the network parameterized by θ. The consistency loss, which is the mean squared error (MSE) between probability distributions, can be given by the following equation:

$$L_{\mathrm{cons}}={∥p-\tilde{p}∥}_{2}^{2}$$

where p and $\tilde{p}$ are the predicted class probability distributions for the original and perturbed images, respectively. $L_{\mathrm{cons}}$ enforces prediction invariance under common spatial transformations.

### 3.8. Training Objective

The general training goal is characterized as a weighted sum of three loss terms:

$$L=L_{\mathrm{CE}}+\lambda_{1}L_{\mathrm{ordinal}}+\lambda_{2}L_{\mathrm{consistency}}$$

where $L_{\mathrm{CE}}$ denotes the cross-entropy loss, $L_{\mathrm{ordinal}}$ denotes the progression-aware ordinal loss, and $L_{\mathrm{consistency}}$ denotes the consistency regularization term. The weighting coefficients $\lambda_{1}$ and $\lambda_{2}$ are chosen empirically to balance classification accuracy, progression awareness, and robustness. The ordinal loss and consistency regularization weighting coefficients were empirically determined to 0.3 and 0.2, respectively, and through initial experimentation these values provided the best tradeoff between classification, progression-aware behavior, and training stability.

### 3.9. Model Interpretability via Token Importance

**Figure 2:**
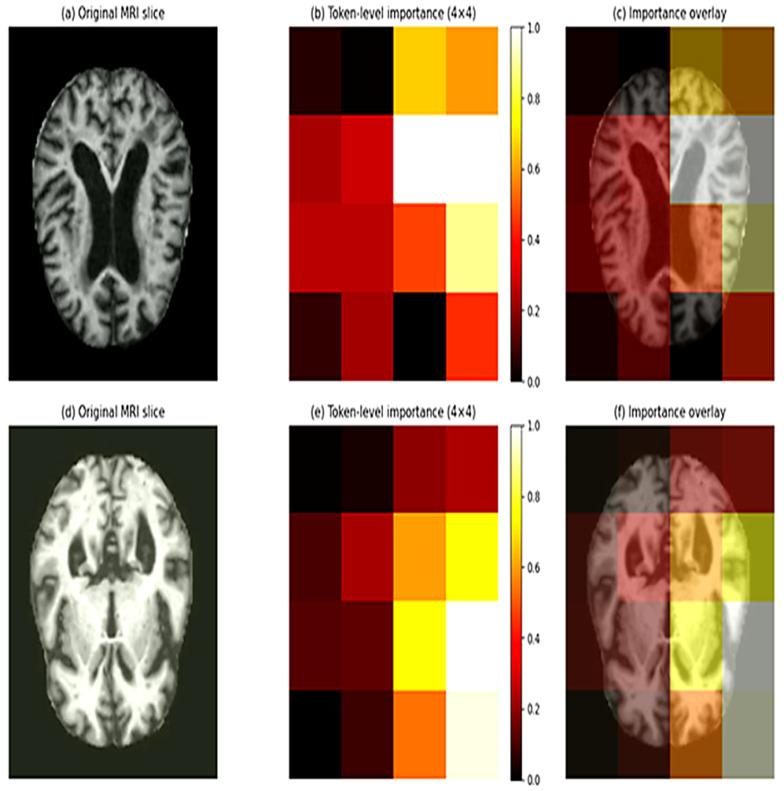
Token-level importance visualization.

In order to enhance interpretability, transformer output embeddings are used to compute token-level importance analysis. The importance of every spatial token is measured by its embedding vector L2 norm. The values of token importance are reconstructed into the form of a spatial map of 4 × 4 and are represented as a heatmap (see Figure 2). The token-level importance maps give an understanding of the spatial regions that most significantly contribute to the stage of disease discrimination, enhancing transparency and credibility to model predictions.

Token-level importance map derived from transformer embeddings. Warmer colors indicate spatial regions that contribute more strongly to Alzheimer’s disease stage classification. The proposed token-level interpretability mechanism of DeepAttentionADNet is shown in Figure 2. Given an input MRI slice, the transformer generates a sequence of spatial token embeddings of a 4 × 4 grid which is acquired through adaptive pooling. The significance of the individual token is estimated by the L2 norm of the embedding vector indicating the relative role of the spatial area in the prediction of the model. These tokens’ importance values are then rearranged as a 4 × 4 spatial map and represented as a heatmap where warmer colors indicate higher relevance. The heatmaps are also superimposed on the original MRI slices to help in the interpretation of the anatomy. According to Figure 2, the model consistently draws attention to areas related to structural brain alterations in relation to the evolution of the Alzheimer disease that gives intuitive understanding of the process of making decisions and adds more transparency and confidence to the proposed framework.

### 3.10. Implementation Details

DeepAttentionADNet framework is proposed and implemented using the PyTorch version 2.9.0 deep learning library. Prior to tokenization, the CNN feature maps are adaptively pooled to a 4 × 4 spatial grid to balance interpretability, computational efficiency, and spatial resolution. By avoiding excessive token fragmentation, which could introduce noise and increase computational overhead, this resolution maintains coarse anatomical structure throughout the MRI slice. 16 spatial tokens are produced by a 4 × 4 grid, which is enough to capture global spatial patterns associated with the progression of Alzheimer’s disease while preserving a consistent and comprehensible token layout for attention-based analysis. Prior vision transformer and hybrid CNN–Transformer studies have demonstrated that this design offers an efficient trade-off between representational capacity and efficiency for medical imaging tasks, allows consistent token-level importance visualization, and makes comparisons across samples and disease stages easier. A single Transformer layer with four attention heads and learnable positional embeddings processes each token after it has been projected to an embedding dimension of 256. Mean pooling is then used for classification. 100 epochs with early stopping is used to train the model is using a batch size of 8, a learning rate of $3\times 10^{-4}$, and a weight decay of $1\times 10^{-4}$. Because it has been demonstrated to enhance generalization in deep neural networks by separating weight decay from gradient-based parameter updates, the AdamW optimizer is used.

Mixed-precision training is used with automatic mixed precision to increase the efficiency of computation and reduce GPU memory, which allows consistent optimization and faster training. This is done through a leakage-free 5-fold cross-validation strategy used to evaluate performance. In each fold, the early stopping is applied based on validation accuracy to mitigate overfitting, with training stopped when no performance improvement is observed after a specified number of epochs. This plan means that the training is done efficiently while preventing over-optimization with the training data. The metrics that are used to comprehensively evaluate the model performance are: the accuracy, precision, recall and F1-score which reflect the effectiveness of the classification across all classes. Besides, the area under the receiver operating characteristic curve (AUROC) and the area under the precision–recall curve (AUPR) are reported to provide a solid measure of discriminative performance. The results are provided as an average performance of all cross-validation folds that are reported to assess the reliability and unbiasedness of the model.

## 4. Results

**Figure 3:**
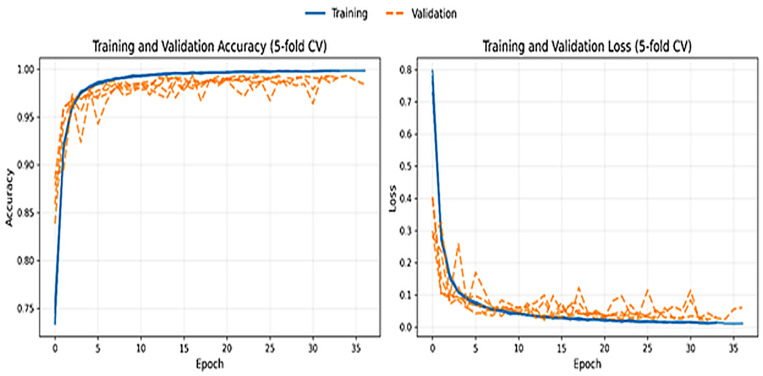
Training and validation performance of DeepAttentionADNet across 5-fold cross-validation.

The averages of the training epoch curves of training and validation accuracy (left) and loss (right) curves are indicated as a function of the training epochs. The training and validation trends are very close which means that there is no overfitting and the generalization is stable. Figure 3 shows the training pattern of DeepAttentionADNet with the 5-fold cross-validation scheme. As illustrated in Figure 3 (left) training and validation accuracy increase rapidly in the first few epochs and then levels off to stay consistently high with only slight variations across the folds. The fact that training and validation accuracy curves are very close implies effective generalization and implies that the model is not overfitting the training data even though the classification performance is very high. This observation is further supported by Figure 3 (right) whereby both training and validation loss decreases, and levels off to low values. Whilst minor oscillations can be observed in the validation loss especially at the early part of the training process, the oscillations gradually reduce with the continued training which indicates a stable convergence across the various folds. All in all, the fact that the trends in training and validation are consistent across different folds proves the efficiency of the proposed optimization strategy and indicates that the established regularization strategies and early stopping parameters are efficient to manage overfitting.

**Figure 4:**
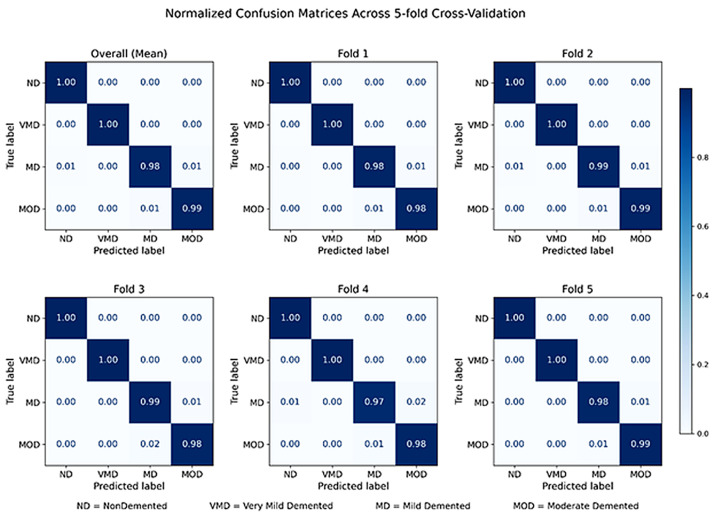
Normalized confusion matrices of DeepAttentionADNet across 5-fold cross-validation.

The performance of DeepAttentionADNet, which is evaluated on a 5-fold cross-validation protocol, is summarized in Table 1. The model has a very high accuracy, precision, recall, and F1-score, and the mean accuracy of the model is 98.97. The standard deviations are low and uniform in all metrics, which implies consistency of performance and high resistance to changes in data splits. It is important to note that the value of the AUROC and the AUPR is very close to the value unity in all folds, which demonstrates that the model has a high level of discrimination and is quite reliable in rating disease stages. The minor change in the performance of Fold 4 is captured in the reported standard deviation but is within an acceptable range, which implies that the model can generalize even with more difficult partitions of data. Altogether, these findings indicate that the proposed framework provides a high classification accuracy, as well as consistent performance across folds, which is why it can be recommended in reliable multiclass Alzheimer’s disease staging. The results of the Cohen’s kappa values obtained on the five cross validation folds are consistently high with a range of 0.9783 to 0.9899 and an average of 0.9861 ± 0.004. These outcomes show that there is almost perfect agreement of the predicted and ground-truth labels. The small standard deviation indicates that the proposed model is stable and robust to variations in the data splits. In general, the high and stable kappa scores also confirm the validity of the proposed multiclass Alzheimer’s disease staging system. The normalized confusion matrices obtained under the 5-fold cross-validation framework are shown in Figure 4, which shows both the overall mean confusion matrix and fold-wise confusion matrices. The average confusion matrix shows a high rate of diagonal dominance which shows high accuracy of classification among all the four stages of the Alzheimer disease. Classes Non-Demented and Very Mild Demented are always classified with near-perfect accuracy, as the model has the capacity to make reliable distinction between cognitively normal subjects and early-stage dementia. Mild and Moderate Demented classes are characterized by minor confusion and are mainly between the adjacent disease stages which would be in tandem with the slight anatomical differences in MRI images with increasing level of disease severity. Noteworthy, there is no significant misclassifications between the stages, which are distant like Non-Demented and Moderate Demented, which supports progress-sensitive behavior of the model. The fold-wise confusion matrices also verify the soundness of the propsed framework, with the same set patterns of classification being constantly in all partitions of the data. Overall, these findings indicate that DeepAttentionADNet can provide reliable and clinically plausible multiclass staging of Alzheimer’s disease and at the same time, the model is robust across cross-validation folds.

**Figure 5:**
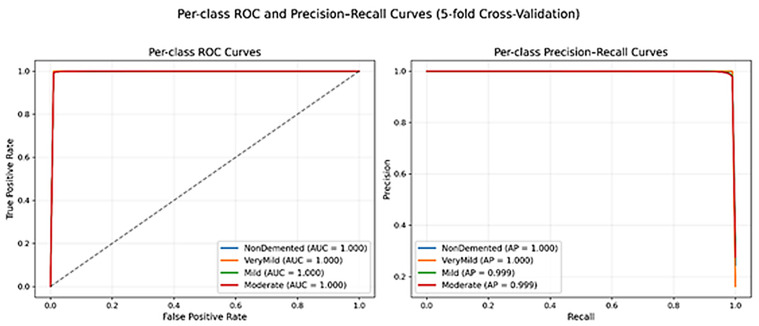
Per-class ROC and precision–recall curves of DeepAttentionADNet under 5-fold cross-validation. Receiver operating characteristic (ROC) curves (**left**) and precision–recall (PR) curves (**right**).

Figure 5 shows the per-class receiver operating characteristic (ROC) as well as precision recall (PR) curves achieved over the 5-fold cross-validation protocol. The ROC curves of all the four stages of Alzheimer disease closely follow the upper-left corner as illustrated in Figure 5 (left) hence near-unity AUC values. It means that the proposed model has a high capability to predict the disease stage against the rest of the classes based on different decision thresholds. This behavior is also verified by the precision-recall curves in Figure 5 (right), which show that the precision is consistently high even in the presence of a wide range of recall values of all classes. It is worth noting that the PR analysis indicates reliable performance even in the conditions when the presence of a class imbalance can affect the precision, which supports the soundness of the proposed framework. Although the curves are closer to the ideal performance, the findings must be viewed along with the cross-validation procedure and the ordinal error analysis. Collectively, such results indicate that DeepAttentionADNet has gained high discriminative performance and still has a clinically consistent prediction behavior across disease stages.

The close near-unity that was obtained in the values of the AUROC and AUPR across folds ought to be viewed within the realms of the dataset nature and the evaluation procedure. The Singhal multiclass MRI data is composed of skull striped, and centered brain slices with less background variability, which has been reported to produce high levels of discriminability in slice-level classification tasks. To reduce the possibility of evaluation leakage, data splitting is carried out in a strict way before the augmentation, where no transformed version of the same image is represented in some folds. Notably, the stability of the performance in folds, low standard deviation of the measures, and the ordinal error statistics indicate that the reported performance could not be an artifact of data leakage but instead that of stable and progression-consistent learning. Computing all reported results at the slice level is due to the absence of subject-level grouping data in the dataset needed to compute aggregation of results on a patient-wise basis. Thus, performance measures record slice-wise discriminability and not ultimate clinical diagnosis.

### 4.1. Ordinal Error Analysis and Clinical Consistency

Correct staging of Alzheimer disease is not only a question of accuracy of classification, but also a question of clinical plausibility because the stages of the disease have a strict ordinal progression. Multiclass accuracy is more traditional, but it does not differentiate between minor and clinically acceptable errors, and severe errors that contradict the progression of the disease. To overcome this weakness, we performed an extensive ordinal error analysis to assess the extent to which the proposed model respects the natural severity ordering of the stages of Alzheimer disease. This analysis provides deeper insight into the behavior of prediction by providing additional understanding of the extent of error in misclassification, both in magnitude and direction, to offer a clinically meaningful analysis of prediction beyond the standard performance measures.

**Figure 6:**
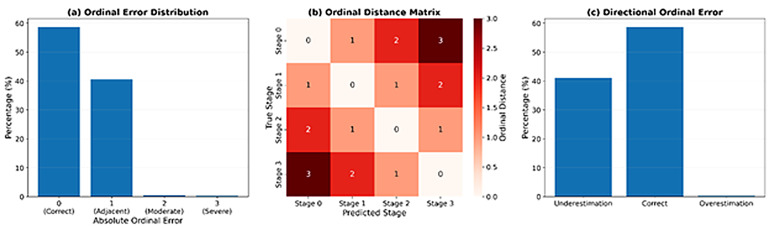
Analysis of Ordinal Prediction Errors and Directionality in Alzheimer’s Disease Staging.

Figure 6 provides a descriptive study of ordinal prediction errors to assess the progression-conscious behavior of the proposed model. Figure 6a shows the distribution of absolute ordinal errors, which are expressed in the form of percentages to eliminate the bias based on the size of the dataset or cross-validation splits. A vast majority of the predictions are correct (Delta = 0), but a large proportion of all errors are restricted to adjacent-stage misclassifications (Delta = 1). Two-stage differences (Delta = 2) are very uncommon, and no serious ordinal errors (Delta = 3) are detected. The absence of large ordinal deviations shows that the model avoids effectively clinically implausible predictions, such as directly placing non-demented subjects on moderate stages of dementia. These findings indicate that the progression-aware approach to learning can be efficiently used to make predictions constrained to realistic disease transitions. To further investigate the structure of ordinal misclassifications, Figure 6b illustrates a distance-aware ordinal matrix, whereby each of the cells displays the ordinal distance between the actual and predicted disease stages. The high density of values in the main diagonal shows the highest percentage of predictions are equal to the ground truth or within one stage. As the distance from the diagonal increases, the error values grow larger, indicating more severe clinical misclassifications. Notably, the fact that there are no high-distance errors is a verification that the model maintains the natural ordering of the stages of the progression of Alzheimer disease and does not make long-range jumps between different stages, which is critical in modeling the progression of neurodegenerative diseases. Figure 6c illustrates directional distribution of ordinal errors by separating underestimation and overestimation propensity. Majority of these samples are rightly classified, and there is more underestimation errors as opposed to overestimation errors. The level of overestimation is low which is the benefit of a clinical situation because it is possible to predict an advanced disease that is clinically favorable because predicting a more advanced disease stage than the true condition may lead to unnecessary psychological burden or premature clinical intervention and this will cause the establishment of an unwarranted psychological load or early clinical intervention. This asymmetric error distribution indicates that the model is conservative in its prediction behavior where it is prioritizing clinically safer misclassifications when uncertainty is present.

### 4.2. Token-Level Interpretability and Disease Progression Analysis

To further improve the interpretability of DeepAttentionADNet as well as to understand how the model uses spatial information on the disease stages, we conduct a detailed token-level importance analysis regarding the transformer embeddings. The importance of tokens is based on the L2 norm of transformer output embeddings and represents the amount of importance of a particular spatial region to the final classification decision. The analysis is done on all the cross-validation folds and covers properties of distribution, sample-wise spatial relevance, stability across ample, and disease-stage behavioral progression. Cumulatively, these analyses can give an understanding of the reliability as well as the clinical plausibility of the learned representations.

**Figure 7:**
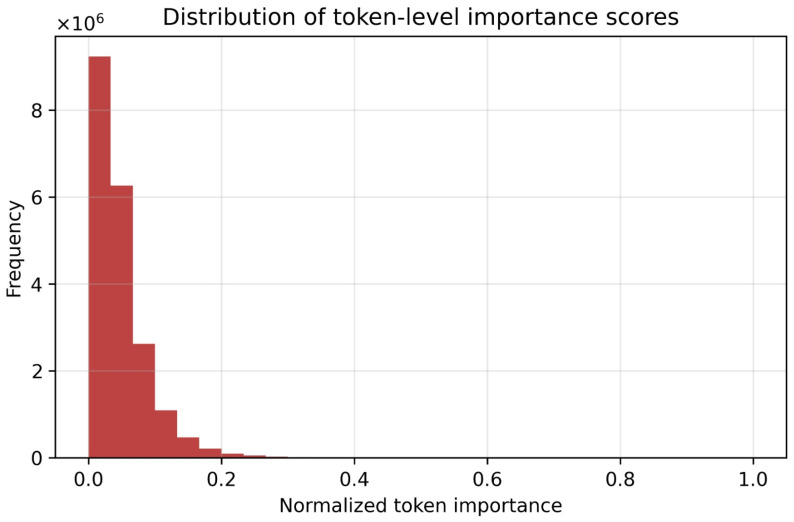
Distribution of normalized token-level importance scores across all samples.

Figure 7 shows the global distribution of normalized token-level importance scores in the whole samples and spatial tokens. The distribution is highly skewed with a few tokens exhibiting high importance and the rest make a slight contribution to the final prediction. This tendency indicates that the model is selective in the distribution of attention to certain spatial locations as opposed to the uniformity of distribution across the image. This sparsity in token significance in medical imaging applications is desirable as it is indicative of significant localization of discriminative anatomical patterns as opposed to diffuse or noisy attention.

**Figure 8:**
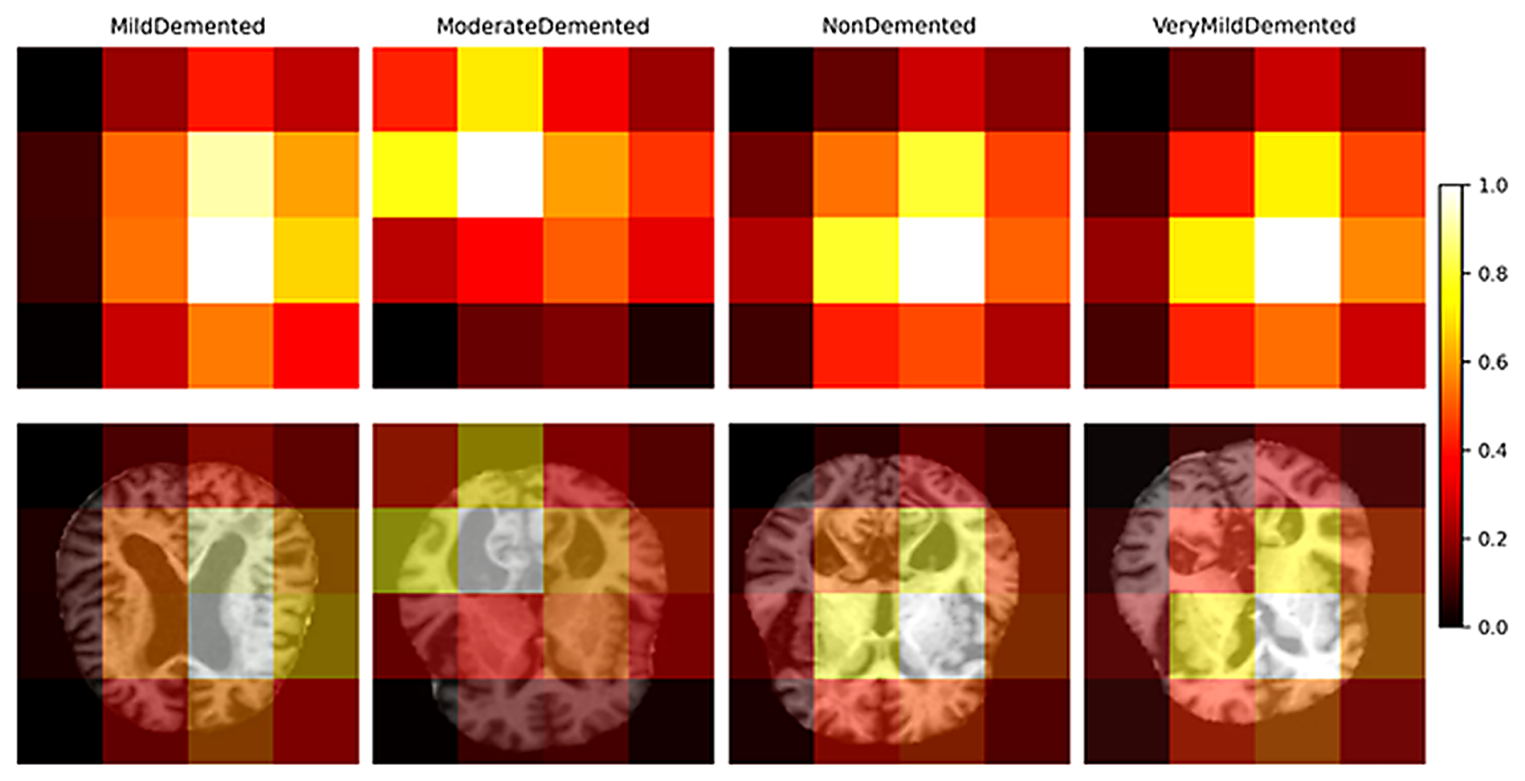
Class-wise average token importance maps and corresponding spatial overlays on MRI slices.

**Figure 9:**
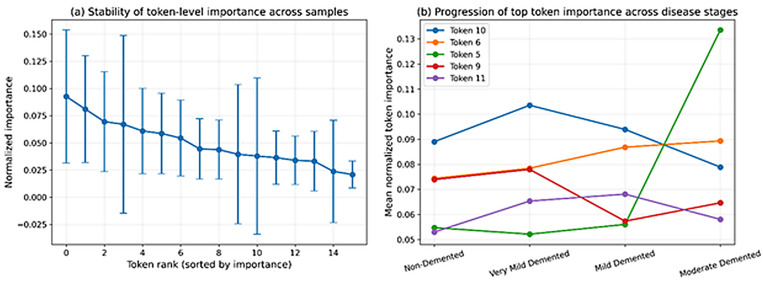
Analysis of token-level importance stability and disease progression. (**a**) Mean and standard deviation of normalized token importance across samples, ranked by global relevance. (**b**) Evolution of the most influential token importance values across Alzheimer’s disease stages.

Figure 8 shows the class-wise average token importance maps per stage of the Alzheimer disease and its corresponding overlays on the representative MRI slices. The heatmaps illustrate that there are specific patterns of spatial attention in disease stages which means that the model pays attention to various sections of the brain based on the severity of the disease. The overlay maps also support interpretations (anatomy) by indicating the proximity of the loaded highly weighted tokens and the underlying brain structures. Such findings indicate that DeepAttentionADNet learns stage-specific spatial representations, which support the interpretability of the proposed framework and its clinical importance. To assess the stability of token-level importance across samples, Figure 9a reports the means and standard deviation of normalized score of importance of tokens ranked by the global relevance. The findings indicate that there is a sharp downward trend in importance with an increase in token rank and the associated variability remains moderate. This means that the most influential tokens are always important across samples, which proves the effectiveness and trustworthiness of the learned representations. Figure 9b also looks at the change in the importance of the most relevant tokens by the stages of the disease. The trajectories portrayed indicate that there are systematic and non-uniform variations in the importance of tokens with increase in disease severity. This indicate that the model captures the spatial patterns of progression rather than static features. The observed trends point to the fact that some spatial areas get informative or less informative with the progression of the Alzheimer disease, which is consistent with the progressive characteristics of neurodegeneration. All these analyses together confirm that the attention mechanism in the model is stable across samples, as well as sensitive to disease progression.

**Figure 10:**
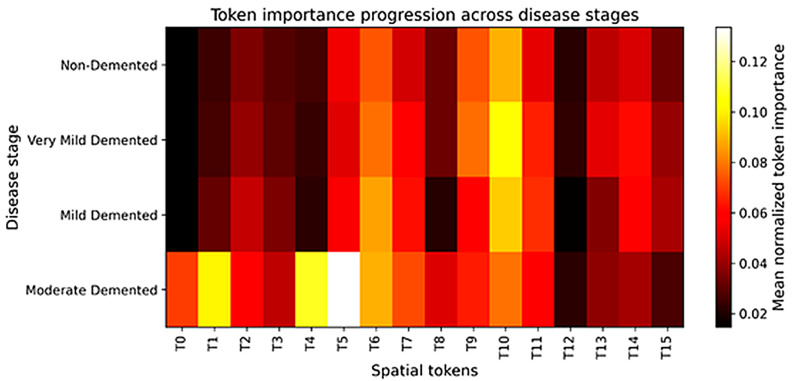
Progression of spatial token importance across Alzheimer’s disease stages.

Figure 10 shows the importance of the spatial tokens learned by the module across the four stages of Alzheimer disease. The rows are associated with the disease stages, and the columns represent the 16 spatial tokens that are obtained following the adaptive pooling and tokenization. The intensity of the colors indicates the contribution of each token to the classification decisions of the model. The figure reveals a clear stage-dependent variation in token importance. The relative token importance is moderate in the non-demented and very mild demented stages and more evenly distributed; thus, indicating that the model depends on a broader set of spatial cues in the event of subtle structural changes. As the disease moves to mild and moderate dementia, several tokens become markedly more significant, which indicates that the model progressively focuses on specific spatial regions that become more discriminative with increasing disease severity. It is important to note that the moderate dementia stage demonstrates the highest concentration of strongly activated tokens indicating the existence of more significant anatomical changes. This gradual increase in the significance of tokens across stages supports the progression-aware design of the proposed framework by showing that the model learns representations that change consistently with the disease severity rather than producing abrupt or clinically unrealistic transitions. Overall, the identified trends prove that DeepAttentionADNet is able to capture stage-sensitive and interpretable spatial representations, which support the appropriateness of the interpretation of multiclass staging of Alzheimer disease and provide further insight into the process that drives the decision of the model.

### 4.3. Ablation Study

An ablation study where we compared pure CNN baseline (ResNet-18) with the entire CNN–Transformer model when they are trained under the same experimental conditions, such as the same data splits, augmentation strategy, loss functions, optimizer, and early stopping criteria was performed to assess the contribution of the Transformer module in the proposed DeepAttentionADNet framework. The CNN-only model had a mean accuracy of 0.9935 with a standard deviation of 0.0019 and F1-score of 0.9943 with a standard deviation of 0.0017, whereas the CNN–Transformer model had a very similar accuracy of 0.9924 with a standard deviation of 0.0020 and an F1-score of 0.9933 with a standard deviation of 0.0019. Those findings indicate that the proposed architecture does not reduce the high level of discriminative performance provided by the convolutional backbone. Notably, the Transformer module gives structured token-level spatial representations, through an explicit globally based connection between spatial regions, which is the basis of the interpretability and disease progression analysis. As a result, the accuracy between the two variants does not differ significantly, but the addition of the Transformer makes the model more transparent, allows attributing relevance with tokens, and makes progression-sensitive spatial interpretation, which are the main goals of the presented work.

## 5. Discussion

This paper proposed DeepAttentionADNet, a progression-aware and interpretable CNN–Transformer framework for multiclass stage Alzheimer disease using MRI images. In contrast to most of the existing methods that classify disease stages as autonomous classes, the presented model directly adds disease severity ranking, robustness constraints, and token interpretability, which helps address some of the most important limitations of the existing literature on the classification of Alzheimer disease. The experimental results prove that DeepAttentionADNet achieves high level of classification accuracy in every fold of a 5-fold cross-validation protocol, without leaking information. The close alignment of training and validation curves as well as low variance across folds demonstrates high generalization and confirms that the reported performance was not a result of overfitting or evaluation leakage. Notably, the adoption of dataset splitting before augmentation is a contributing factor to the unbiased evaluation that is commonly overlooked in previous studies. In addition to the standard performance measures, the ordinal error analysis is used to gain more insight into the clinical plausibility of the model predictions. Conventional accuracy measures put emphasis on all misclassifications, whereas the staging of Alzheimer’s disease has an ordinal progression. The results indicate most of the incorrect predictions resulted from the adjacent disease stages, and no long-term stage misclassifications were observed. This result shows that the progression-aware ordinal loss is effective to limit predictions to realistic disease transitions with fewer highly implausible results like direct misclassification between non-demented and moderate dementia. Additionally, the directional error analysis indicates that there is a low rate of overestimation errors with a conservative prediction tendency which is good in clinical decision-support systems since overestimation of disease severity can result in unnecessary interventions. These results are further supported by the confusion matrix analysis which shows that there is strong diagonal dominance in all folds. In cases of misclassifications, they are mostly within stages adjacent to each other, as they are very subtly anatomically different, and this is the characteristic of the early development of Alzheimer disease. The absence of overlap in the stages of the disease at a distance proves that the model respects the natural ordering of disease severity and captures meaningful structural distinctions rather than spurious correlations. The transformer-based tokenization mechanism is also vital since it allows defining the current work in terms of its interpretability analysis. The token-level importance distribution proves that the model is based on a sparse subsample of spatial tokens, implying selective attention to discriminative brain regions rather than diffuse or noisy feature utilization. Class-wise importance maps, as well as the overlay visualizations, also indicate stage-specific spatial patterns, highlighting how attention shifts as disease severity increases. Stability analysis assures that the most influential tokens are always significant across samples indicating robust and reliable representations. Importantly, the importance analysis of progression of token demonstrates systematic changes in spatial relevance across disease stages. At earlier stages, the attention pattern is more distributed whereas at later stages, the token activations are stronger and more localized. This progressive change is consistent with the established pattern of neurodegenerative changes of Alzheimer’s disease, and this further demonstrates that DeepAttentionADNet is learning progressive-aware representations, and not static classification signals.

To measure this behavior, we determined a token concentration score of each slice, which is the ratio of overall token significance that is explained by the most significant spatial tokens. The findings demonstrate that the concentration of attention increases monotonically with disease severity and thus the model is increasingly concentrated on a smaller number of discriminative regions as the Alzheimer disease progresses. In addition to that, the correlation between disease stage index and token concentration is positive, which gives quantitative evidence that token-level relevance increases systematically with disease stage and is congruent with known imaging biomarkers of Alzheimer disease which become increasingly apparent at later stages.

From a methodological perspective, by integrating a lightweight transformer module, the model will be able to capture long-range spatial correlations that can be challenging to model based on convolutional operations alone. Together with consistency regularization, this design is robust to typical MRI perturbation and perform better at slice-level stability. Importantly, these enhancements are realized without the need of multimodal data or complicated preprocessing pipelines, so the proposed framework is more feasible to be applied in the clinical context in the real world.

Clinically, the proposed DeepAttentionADNet system can be incorporated into radiological routine and used as a decision support tool, not a diagnostic system. Practically, MRI images obtained as a result of regular neuroimaging procedures would be processed in the conventional way (e.g., skull stripping and slice extraction) first and sent to the trained model as part of a post-acquisition analysis pipeline. The model can generate slice level predictions which can be formed into subject level predictions through probabilistic pooling or majority voting to come up with a final disease stage prediction. The results can be displayed to radiologists as overlay heatmaps and a score of confidence which can be presented as complementary to clinical decision, using existing picture archiving and communication systems. The token-level importance maps can give spatial explanations which could be reviewed with MRI slices so that the clinicians could evaluate the model attention of known regions attacked by Alzheimer disease. This interpretability is what is critical to trust and regulatory acceptance and eventual implementation in clinical decision-support environments. Notably, the lightweight design and slice-based processing allow to achieve high inference rates, so the framework could be deployed successfully to real-time or near-real-time clinical workflows.

Although the study has a good performance, Theres are limitations that need to be acknowledged. First, the model is tested on one, balanced multiclass data and even though leakage-free test is imposed, additional testing on independent data and multi-center cohort would reinforce the generalizability arguments. Moreover, token-level interpretability can be used to give spatial insight, and it does not replace voxel-level or region-of-interest-based clinical interpretation. Third, although slice-level analysis makes fine-scale analysis of spatial patterns possible, and allows the token analysis to be interpreted at the subject clinical diagnosis, it is not directly applicable to subject-level clinical diagnosis. This is a limitation of the two-dimensional MRI slices utilized since there is no explicit modeling of inter-slice continuity or inter-slice volumetric disease patterns that define the progression of Alzheimer disease. Practically, subject-level prediction can be acquired by voting or probabilistic pooling strategy, which will be examined in future studies. It is interesting to note that the ordinal error analysis shows that the misclassifications are mostly restricted to neighboring disease stages which imply clinically plausible behavior even at the slice level. This property is needed in the case of aggregating predictions at the subject level because it minimizes the possibility of serious diagnostic errors. Fourth, Future work may extend this framework to volumetric MRI analysis, longitudinal disease progression modeling, and integration with clinical or cognitive biomarkers.

## 6. Conclusions

In this paper we propose DeepAttentionADNet, a progression-aware and interpretable CNN–Transformer architecture for multiclass staging of Alzheimer disease based on MRI images. The model clearly incorporates the disease severity ordering by using an ordinal learning objective, increases the robustness by using consistency regularization, and enhances the transparency by analyzing the token-level interpretability. A leakage-free cross of validation protocol provides fair performance assessment and reliable generalization. Extensive experiments show that the proposed framework obtains high and stable performance across disease stages and can make clinically consistent predictions without interfering with the natural progression of Alzheimer disease. Ordinal error analysis confirms that misclassifications are found in most cases to be determined by adjacent stages, avoiding clinically implausible outcomes. Moreover, analysis of interpretability shows significant stage-specific spatial attention patterns, which change with the severity of the disease, and support the plausibility of model decisions In general, DeepAttentionADNet is a strong and clinically compatible system of multiclass staging of Alzheimer disease. This work will make a significant contribution to the development of reliable decision support systems based on deep learning as an effective aid in the diagnosis and management of neurodegenerative diseases because factors such as accuracy, progression awareness, robustness and interpretability are collectively addressed.

## Figures and Tables

**Table 1:** Performance of DeepAttentionADNet under 5-fold cross-validation.

Fold	Accuracy	Precision	Recall	F1-Score	AUROC	AUPR	Cohen’s Kappa
Fold 1	0.9901	0.9912	0.9915	0.9914	0.9998	0.9995	0.9866
Fold 2	0.9926	0.9932	0.9936	0.9934	0.9999	0.9998	0.9899
Fold 3	0.9908	0.9919	0.9920	0.9920	0.9998	0.9996	0.9876
Fold 4	0.9840	0.9854	0.9869	0.9861	0.9994	0.9988	0.9783
Fold 5	0.9912	0.9919	0.9927	0.9923	0.9998	0.9996	0.9881
**Mean**	**0.9897**	**0.9907**	**0.9913**	**0.9910**	**0.9998**	**0.9994**	**0.9861**
**Std**	**0.0033**	**0.0031**	**0.0026**	**0.0028**	**0.0002**	**0.0004**	**0.0040**

## Data Availability

Suggested Data Availability Statements are available at https://www.kaggle.com/datasets/uraninjo/augmented-alzheimer-mri-dataset-v2 (accessed on 2 January 2026).
